# Approaches for Genetic Discoveries in the Skin Commensal and Pathogenic *Malassezia* Yeasts

**DOI:** 10.3389/fcimb.2020.00393

**Published:** 2020-08-07

**Authors:** Giuseppe Ianiri, Joseph Heitman

**Affiliations:** ^1^Department of Agricultural, Environmental and Food Sciences, Università degli Studi del Molise, Campobasso, Italy; ^2^Department of Molecular Genetics and Microbiology, Duke University Medical Center, Durham, NC, United States

**Keywords:** *Malassezia*, genomics, *Agrobacterium tumefaciens*-mediated transformation (AMT), insertional mutagenesis, targeted gene replacement

## Abstract

*Malassezia* includes yeasts belong to the subphylum Ustilaginomycotina within the Basidiomycota. *Malassezia* yeasts are commonly found as commensals on human and animal skin. Nevertheless, *Malassezia* species are also associated with several skin disorders, such as dandruff/seborrheic dermatitis, atopic eczema, pityriasis versicolor, and folliculitis. More recently, associations of *Malassezia* with Crohn's disease, pancreatic ductal adenocarcinoma, and cystic fibrosis pulmonary exacerbation have been reported. The increasing availability of genomic and molecular tools have played a crucial role in understanding the genetic basis of *Malassezia* commensalism and pathogenicity. In the present review we report genomics advances in *Malassezia* highlighting unique features that potentially impacted *Malassezia* biology and host adaptation. Furthermore, we describe the recently developed protocols for *Agrobacterium tumefaciens*-mediated transformation in *Malassezia*, and their applications for random insertional mutagenesis or targeted gene replacement strategies.

## *Malassezia* Yeasts as Commensals and Pathogens

*Malassezia* includes a monophyletic genus of yeasts that are the main fungal species resident on human skin and hair, representing more than 90% of the eukaryotic components of the skin microbiome (Findley et al., 2013). To date, 18 species of *Malassezia* have been identified (Theelen et al., 2018). The limited number of species isolated so far most likely reflects the difficulties in cultivating *Malassezia* under laboratory conditions, given their ability to grow *in vitro* only in the presence of exogenous lipids, and at a narrow range of temperatures. As commensal organisms living on the skin, *Malassezia globosa, Malassezia restricta*, and *Malassezia sympodialis* are the most common species found in humans, followed by *Malassezia furfur, Malassezia yamatoensis, Malassezia dermatis, Malassezia obtusa, Malassezia japonica*, and *Malassezia arunalokei*. *Malassezia pachydermatis* is mainly found in dogs and cats, *Malassezia slooffiae* in pigs and cats, *Malassezia nana* in cats and horses, *Malassezia caprae* in goats, *Malassezia equina* in horses, *Malassezia cuniculi* in rabbits, *Malassezia brasiliensis* and *Malassezia psittaci* in parrots, and *Malassezia vespertiliones* in hibernating bats (Theelen et al., 2018; Guillot and Bond, 2020). Aside from their commensal lifestyle, *Malassezia* yeasts are associated with a number of skin disorders, the most common of which are dandruff/seborrheic dermatitis, atopic eczema, pityriasis versicolor, and folliculitis. Occasionally, in immunocompromized hosts or patients receiving total parenteral nutrition, *M. furfur, M. sympodialis*, and *M. pachydermatis* can also cause systemic disease (Gaitanis et al., 2012; Saunders et al., 2012; Velegraki et al., 2015; Theelen et al., 2018; Guillot and Bond, 2020). Moreover, novel studies have linked *Malassezia* yeasts with Crohn's disease in patients with an S12N polymorphism in the gene encoding CARD9, a signaling adaptor critical for innate antifungal immunity (Limon et al., 2019), with pathogenesis of pancreatic ductal adenocarcinoma through activation of the MBL pathway (Aykut et al., 2019), and with cystic fibrosis pulmonary exacerbation (Soret et al., 2020).

## Evolutionary Trajectory of *Malassezia* Genomes Correlates With Pathogenicity and Niche Adaptation

In the last decade several groups contributed to generate genomics data for the majority of species within the *Malassezia* genus. A GenBank search (last accessed on March 7th, 2020) finds 45 genome assemblies that include 15 known *Malassezia* species (Xu et al., 2007; Gioti et al., 2013; Triana et al., 2015; Wu et al., 2015; Park et al., 2017; Zhu et al., 2017; Lorch et al., 2018; Cho et al., 2019; Morand et al., 2019; Sankaranarayanan et al., 2020). Analysis of the genomes available contributed to resolve *Malassezia* taxonomy, and shed light on the evolutionary trajectory of pathogenesis and niche adaptation of this unusual fungal genus.

**Figure 1:**
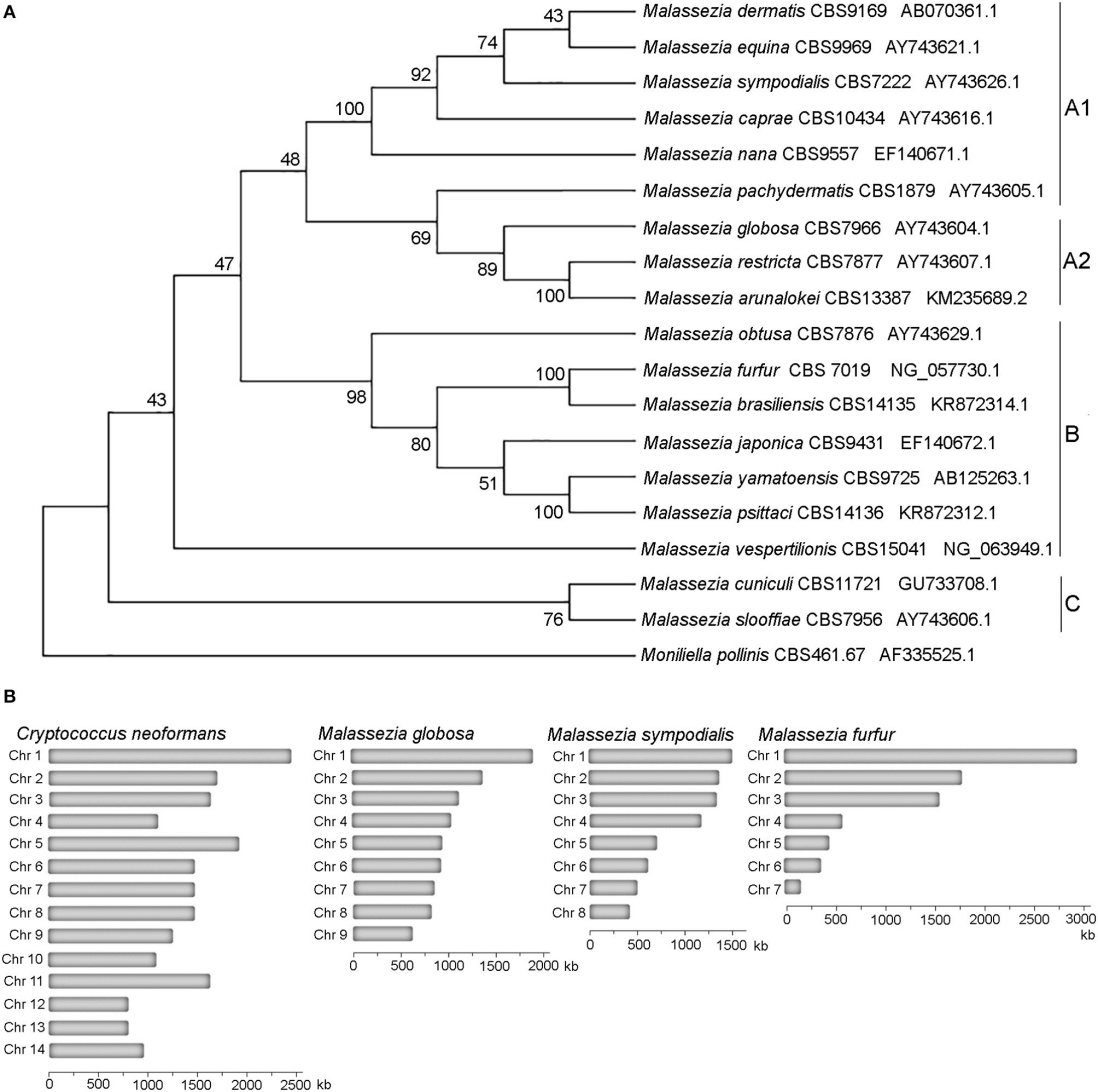
Phylogeny of *Malassezia* yeasts. **(A)** Topology of the type strains of the eighteen recognized *Malassezia* species. The evolutionary history using sequences of the D1D2 domains of the LSU rRNA gene was inferred by using the Maximum Likelihood method based on the Kimura 2-parameter model. The tree with the highest log likelihood (−3126.28) is shown. The percentage of trees in which the associated taxa clustered together is shown next to the branches. Initial tree(s) for the heuristic search were obtained automatically by applying Neighbor-Join and BioNJ algorithms to a matrix of pairwise distances estimated using the Maximum Composite Likelihood (MCL) approach, and then selecting the topology with superior log likelihood value. A discrete Gamma distribution was used to model evolutionary rate differences among sites [5 categories (+G, parameter = 0.1690)]. The rate variation model allowed for some sites to be evolutionarily invariable [(+I), 27.23% sites]. The analysis involved 19 nucleotide sequences. There were a total of 718 positions in the final dataset. **(B)** Karyotypes of representative *Malassezia* species with 9, 8, and 7 chromosomes compared to the Basidiomycete human pathogen *C. neoformans*.

Taxonomically *Malassezia* are included in the subdivision Ustilaginomycotina within the Basidiomycota phylum, which also includes human and plant pathogens (Wang et al., 2014; Wu et al., 2015). Surprisingly, from a phylogenetic viewpoint *Malassezia* fungi are more closely related to the basidiomycete plant pathogen *Ustilago maydis* than the human pathogen *Cryptococcus neoformans*, and are very divergent from other fungi that are found on the skin, such as the dermatophytes and *Candida albicans* (Xu et al., 2007; Saunders et al., 2012; Wu et al., 2015). Within the *Malassezia* genus we found three clades that include two sister clades, clade A and clade B, with clade A including subclades A1 and A2, and clade C that includes early-divergent species (Figure 1A). Phylogenetic relationships of the tree of Figure 1A based on D1D2 domains of LSU rDNA agree with the previous phylogenomics data (Wu et al., 2015; de Hoog et al., 2017; Lorch et al., 2018; Theelen et al., 2018).

All haploid *Malassezia* species have small and compact genomes compared to other phylogenetically related fungi (7–9 Mb compared to ~20 Mb) (Figure 1B), with genes being arranged very close to each other, and containing very short introns. At the karyotype level, haploid *Malassezia* species have from 6 to 9 chromosomes, based on pulsed-field gel electrophoresis (PFGE) and telomere-to-telomere genome assemblies generated with PacBio long-read sequencing technology (Boekhout and Bosboom, 1994; Boekhout et al., 1998; Sankaranarayanan et al., 2020). Using a combination of genomics, biochemical, cell biology, and molecular genetics techniques (described later in the text), Sankaranarayanan and colleagues elucidated the mechanisms of karyotype evolution within the *Malassezia* genus. In particular, the authors proposed an ancestral state of 9 chromosomes and two distinct mechanisms of chromosome number reduction that involve newly-identified AT-rich, fragile, centromeres: a chromosome breakage followed by loss of centromere that gave rise to 8 chromosomes in *M. sympodialis* and closely related species; and centromere inactivation accompanied by changes in DNA sequence following chromosome-chromosome fusion that gave rise to 7 chromosomes in *M. furfur* (Sankaranarayanan et al., 2020). It is intriguing to note that species with 9 chromosomes, such as *M. globosa* and *M. restricta*, are difficult to isolate and replicate in axenic conditions, while *M. sympodialis* and *M. furfur* are more readily cultivated.

**Figure 2:**
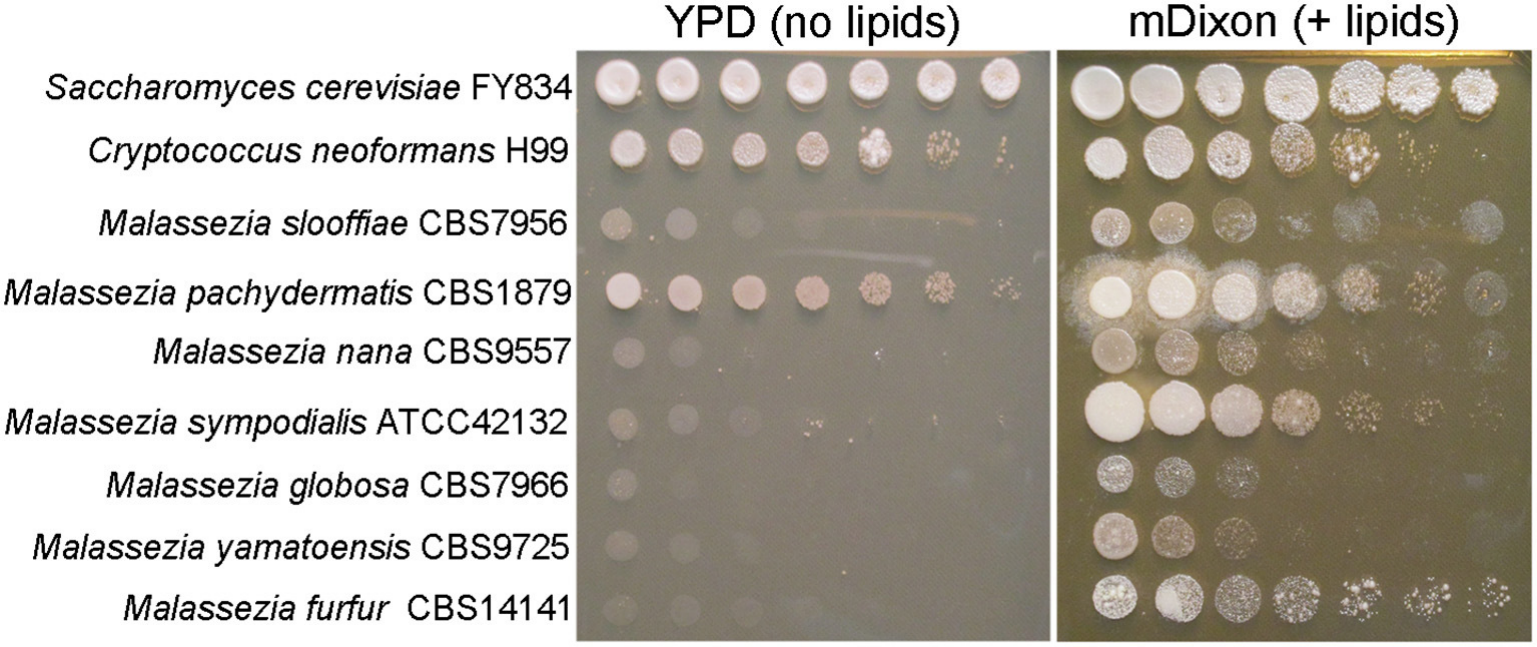
*Malassezia* yeasts are lipid dependent. Ten-fold serial dilution of representative *Malassezia* species on medium without exogenous lipids (YPD, yeast extract 10 g/L, peptone 20 g/L, dextrose 20 g/L, agar 20 g/L), and on lipid-rich medium mDixon (36 g/L malt extract, 10 g/L desiccated ox-bile, 10 g/L mycological peptone, 2 ml/L glycerol, 10 ml/L Tween 60, agar 20 g/L).

At the gene level, comparative genomics revealed extensive turnover events, with significant gene loss and gene gain. Some *Malassezia* species have lost nearly 800 genes and have <4,000 predicted genes. All species have lost genes for lipid metabolism, including fatty acid synthase, Δ9-desaturase, and Δ^2,3^ -enoyl-CoA isomerase, hence explaining *Malassezia* lipid dependency (Figure 2); *M. pachydermatis* has also lost the genes for lipid metabolism but is the only known *Malassezia* species that is able to grow *in vitro* without the addition of exogenous lipids (Figure 2); however, a recent study identified some *M. pachydermatis* isolates that are unable to grow in synthetic medium without lipids (Puig et al., 2017). Other major groups of lost genes include those encoding glycosyl hydrolases and enzymes involved in carbohydrate metabolism, concordant with the evolution of a skin-adapted fungus that uses lipids as carbon sources. Moreover, the *Malassezia* genomes have a low density of transposable elements, and they lack core genes of the RNA interference (RNAi) pathway, such as dicer, argonaute, and RNA-dependent RNA polymerase.

Because the lack of the RNAi pathway in other fungi such as *Saccharomyces cerevisiae* and *U. maydis* is associated with the presence of dsRNA viruses (Drinnenberg et al., 2011), it was hypothesized that *Malassezia* species could also harbor mycoviruses. Corroborating this hypothesis, dsRNA mycoviruses of the Totiviridae family were found in *M. sympodialis, M. globosa, M. obtusa, M. pachydermatis, M. yamatoensis*, and *M. restricta* (Clancey et al., 2019; Park et al., 2019). In *M. sympodialis*, the viral genome includes two dsRNA elements, one of 4.6 kb that encodes an RNA-dependent RNA polymerase and a capsid protein, and one of 1.4 kb that encodes a novel unknown protein predicted to be secreted from the fungal cells and involved in host-pathogen and/or microbial interactions. Fungal cells can be cured of the mycovirus upon exposure to high temperature. Transcriptomic analysis of infected and cured strain pairs revealed that the presence of the mycovirus strongly enhances the expression of ribosomal genes, suggesting that the virus conscripts the *Malassezia* transcription and protein synthesis machineries. Lastly, the presence of the *Malassezia* mycovirus correlated with higher pathogenicity in *ex vivo* models (Clancey et al., 2019; Park et al., 2019).

With respect to gene gain, several unique events found in *Malassezia* genomes warrant consideration. First, a set of 44 *Malassezia*-specific gene clusters was identified, but unfortunately most of them have unknown functions that could not be predicted through bioinformatics analyses (Wu et al., 2015). One gene gain event that Wu and colleagues described regarded a gene with a PF06742 domain of unknown function. This gene is conserved in all *Malassezia* species and is absent in all Basidiomycota, suggesting its acquisition by a *Malassezia* ancestor and an important role in *Malassezia* evolution (Wu et al., 2015).

Second, *Malassezia* genomes are characterized by a significant expansion of lipase, phospholipase, peptidase, and protease gene family-encoding products predicted to break down lipids and proteins for growth, and to play roles in host and microbial interactions (Wu et al., 2015). Intriguingly, a similar set of enzymes is found in the genome of *C. albicans*, a phylogenetically distant fungus that also lives on the skin, suggesting an important role in skin colonization and niche adaptation. Moreover, analysis of the *M. sympodialis* and *M. globosa* genomes identified 89 and 169 predicted secreted proteins, most of them without any domain (Schuster et al., 2018). These predicted secreted proteins include several MalaS allergens, such as MalaS1, a β-propeller-folded protein that has fungal orthologs/homologs in some basidiomycetes and ascomycetes (Vilhelmsson et al., 2007; Gioti et al., 2013), MalaS12 that is similar to other fungal GMC oxidoreductases (Zargari et al., 2007) that play diverse roles in fungi, such as mycotoxin biosynthesis in species of *Aspergillus* and *Penicillium* (Tannous et al., 2017), and MalaS7 (in 3 copies) and MalaS8, both of which are *Malassezia*-specific and have unknown predicted roles (Gioti et al., 2013). Besides these, *M. sympodialis* has genes encoding six additional MalaS allergens that are conserved proteins that share high similarity with the corresponding mammalian homologs, and hence can potentially cross-react with T cells and induce skin inflammation (Glatz et al., 2015).

Another characteristic of *Malassezia* genomes is the presence of bacterial genes acquired through horizontal gene transfer (HGT) events. While the number of these events is usually limited, in *Malassezia* more than 30 HGT have been identified (Wu et al., 2015; Ianiri et al., 2020). HGT candidates found in the majority of the *Malassezia* species include genes involved in broad stress resistance, such as flavohemoglobin, catalase, and oxidoreductases, found in some cases in multiple copies. An interesting HGT candidate is the gene encoding a septicolysin-like protein, which is known as a pore-forming bacterial toxin that might play a role as virulence factor (Beceiro et al., 2013; Mosqueda et al., 2014). This gene is absent in all *Malassezia* species phylogenetically related to *M. sympodialis*, and is present in five copies in *M. globosa*. Other acquired genes encode a variety of proteins with different functions, such as hydrolysis, protein transport and folding, and detoxification of xenobiotics (Ianiri et al., 2020).

Using molecular techniques described in the section *Agrobacterium tumefaciens*-Mediated Transformation Enables Insertional Mutagenesis and Targeted Gene Deletion in *Malassezia*, we demonstrated that the HGT of the bacterial flavohemoglobin in *Malassezia* resulted in a gain of function critical for resistance to nitrosative stress and nitric oxide (NO) detoxification (Ianiri et al., 2020). Analysis of the available *Malassezia* genomes revealed additional HGT of another flavohemoglobin-encoding gene that originated from different donor bacteria. Endogenous accumulation of NO in the flavohemoglobin mutant results in downregulation of the allergen-encoding genes, and accordingly, we found that flavohemoglobin has a dispensable role for *Malassezia* pathogenesis. This study represents the first functional analysis of an HGT-acquired gene in *Malassezia*, and the first evaluation of a *Malassezia* mutant in a novel murine skin model (Sparber and LeibundGut-Landmann, 2019; Sparber et al., 2019) to assess the involvement of a *Malassezia* gene in pathogenesis.

## *Agrobacterium tumefaciens*-Mediated Transformation Enables Insertional Mutagenesis and Targeted Gene Deletion in *Malassezia*

Although the availability of sequenced genomes revealed insights about *Malassezia* evolution, adaptation, and gene turnover, the function of specific genes could not be studied because of the lack of transformation systems. Genetic transformation in fungi can be carried out through the combined use of lithium acetate (LiAc) and polyethylene glycol (PEG), biolistic bombardment, electroporation of intact cells or protoplasts, or *A. tumefaciens*-mediated transformation (AMT). We tested the effectiveness of these four techniques to successfully transform *Malassezia*, but despite several attempts, AMT was the only technique that allowed the generation of stable transformants of *M. furfur, M. sympodialis*, and *M. pachydermatis* (Ianiri et al., 2016; Celis et al., 2017).

*A. tumefaciens* is a soil-borne bacterium that has the ability to infect plants to cause a crown gall disease. The infective process is unique and relies on the natural ability of *A. tumefaciens* to genetically engineer host plants by introducing a short DNA fragment into their genome. The DNA fragment is called T-DNA (transfer DNA) and its excision is enabled by virulence proteins induced by acetosyringone, a chemical compound that is produced by wounded plant roots and that attracts *A. tumefaciens*. The T-DNA contains genes that encode products that mimic plant hormones, and once integrated in the host genome, causes an undifferentiated growth of the plant tissues forming a tumor or gall. Researchers have exploited this natural genetic ability of *A. tumefaciens* to transfer a desired DNA molecule, usually a gene marker, into a variety of eukaryotic organisms, such as plants, animal cells, oomycetes, and fungi.

**Figure 3:**
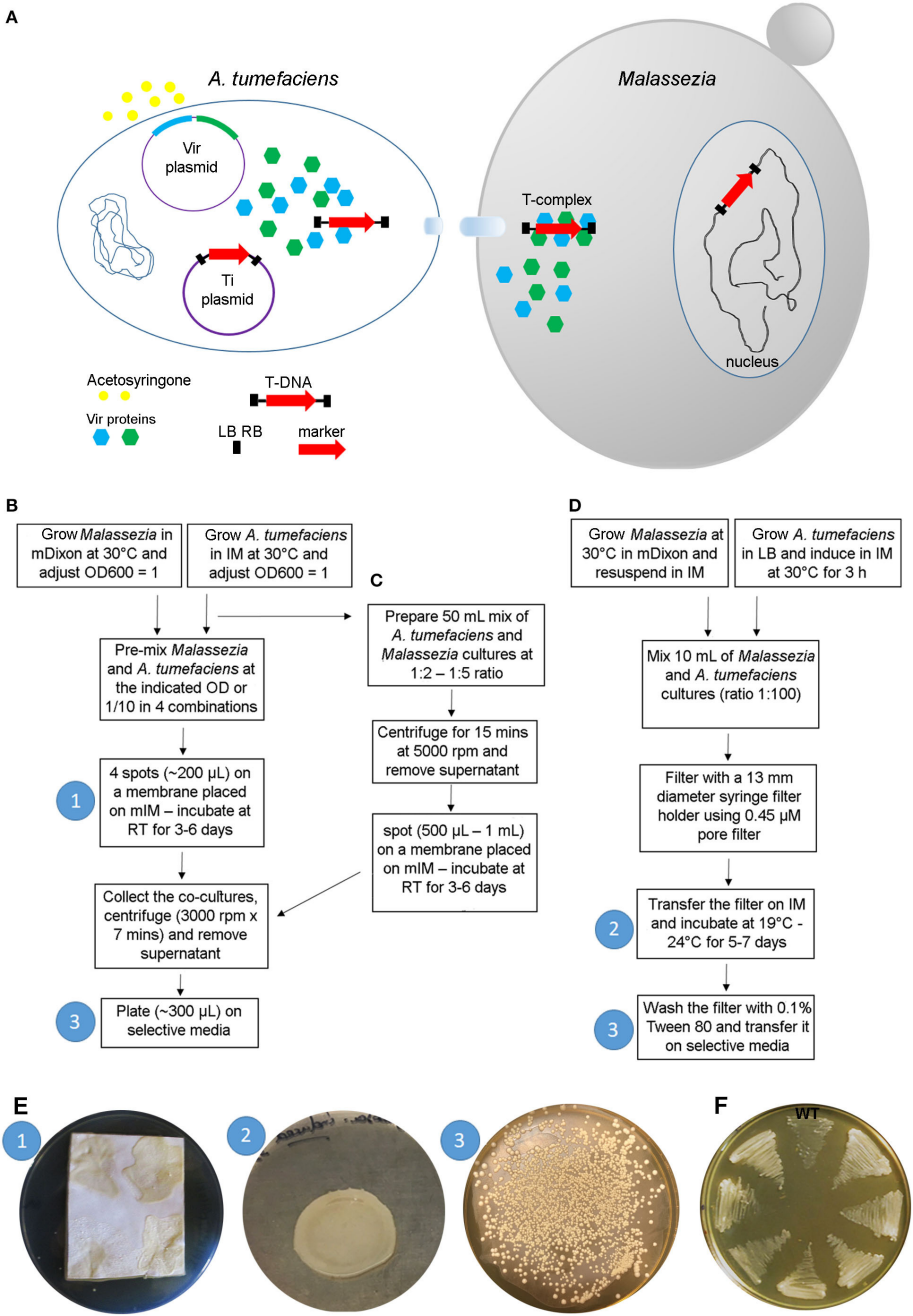
*A. tumefaciens*-mediated transformation of *Malassezia*. **(A)** Schematic overview of the transformation process; see text for details. **(B–D)** Main steps of the protocols available for AMT of *Malassezia* according to Ianiri et al. (2016) **(B)**, Ianiri et al. (2019) **(C)**, and Celis et al. (2017) **(D)**; the white numbers in the blue circle reflect the corresponding step shown in images in **(E)**. **(E)** Representative pictures of the *Malassezia*-*A. tumefaciens* co-incubation step (1) as described in Ianiri et al. (2016), (note the non-homogenous spots due to the presence of Tween that altered the physical proprieties of the IM agar) and (2) in Celis et al. (2017), and example of a highly efficient AMT of *M. furfur* with selection on NAT (3); the white numbers in the blue circles reflect the corresponding steps displayed in the charts shown in **(B–D)**. **(F)** Growth of 7 representatives NAT-resistant M. furfur transformants on NAT selective media compared to the *M. furfur* WT strain.

The most common use of AMT in fungal research is based on a binary vector system: one *A. tumefaciens* plasmid contains *vir* genes required for virulence (i.e., transfer of DNA into the host), and another plasmid, the Ti (tumor inducing) plasmid—usually it is a binary vector and is the most commonly manipulated by researchers—contains the marker gene between two 25-bp direct repeats (right and left borders, RB and LB, respectively) that define the T-DNA. The *vir* proteins are induced by acetosyringone and act on the T-DNA borders enabling the production of single-stranded DNA. The T-DNA is coated with proteins forming the T-complex, which is transferred into the fungal cell. The T-complex is then disassembled, and nuclear localization signals drive the translocation of the T-DNA within the fungal nucleus where integration into the genome occurs (Michielse et al., 2005) (Figure 3A). Compared to other transformation methods, AMT requires basic reagents that are common in most microbiology laboratories, and therefore it has been largely utilized for transformation of yeasts and fungi since its first use in *S. cerevisiae* in 1995 (Bundock et al., 1995). For more information about the method and its use in fungal biology research, there are several reviews available (Michielse et al., 2005; Frandsen, 2011; Idnurm et al., 2017).

In general, the method is straightforward: after growing the *A. tumefaciens* with the binary vector of interest and the fungal strain to be transformed, these two organisms are co-cultured on induction medium (IM) for a few days depending on the growth of the fungus, and subsequently transferred to a selective medium that differs based on the gene marker used (usually a dominant gene that confers resistance to an antifungal drug). A key role in the transformation process is played by the induction medium (IM), which contains acetosyringone to induce the *vir* genes, and it physically supports the *A. tumefaciens-*fungus co-culture ensuring the tight contact between the cells, which is a critical requirement for the success of the trans-kingdom conjugation process (Michielse et al., 2005).

While AMT is relatively simple in the majority of fungi, its use in *Malassezia* turned out to be more difficult because of the unique biology of this fungus. The first successful application of AMT was carried out in *M. furfur*, one of the species that displays more robust growth compared to others within the *Malassezia* genus (Ianiri et al., 2016). The method employed followed a previously published protocol (Ianiri et al., 2011), with the only difference being the use of a modified IM (mIM) that also included exogenous lipids (i.e., Tween and ox-bile) to favor growth of *Malassezia*. Stable *Malassezia* transformants could be generated for the first time, although the efficiency of the ATM was very low (<5 transformants per transformation plate). The AMT method was then improved using a higher density of *Malassezia* cells, a longer co-incubation period of up to 6 days, and by performing the co-incubation step on slightly concave spots generated on nylon membranes placed on the modified IM. The latter modification was critical to facilitate cell-to-cell contact between bacterial and *Malassezia* cells, which was otherwise hindered by the presence of Tween in the modified IM. Subsequently, the AMT protocol was further optimized by Celis et al. (2017) and Ianiri et al. (2019), as illustrated in detail in the flow charts of Figures 3B–D. Examples of representative steps of the AMT of *Malassezia* are shown in Figure 3E, and representative NAT-resistant *M. furfur* transformant are shown in Figure 3F. Lastly, we could never obtain transformants for *M. globosa*, a species characterized by very slow growth at a limited range of temperatures (30–34°C) (unpublished data).

**Figure 4:**
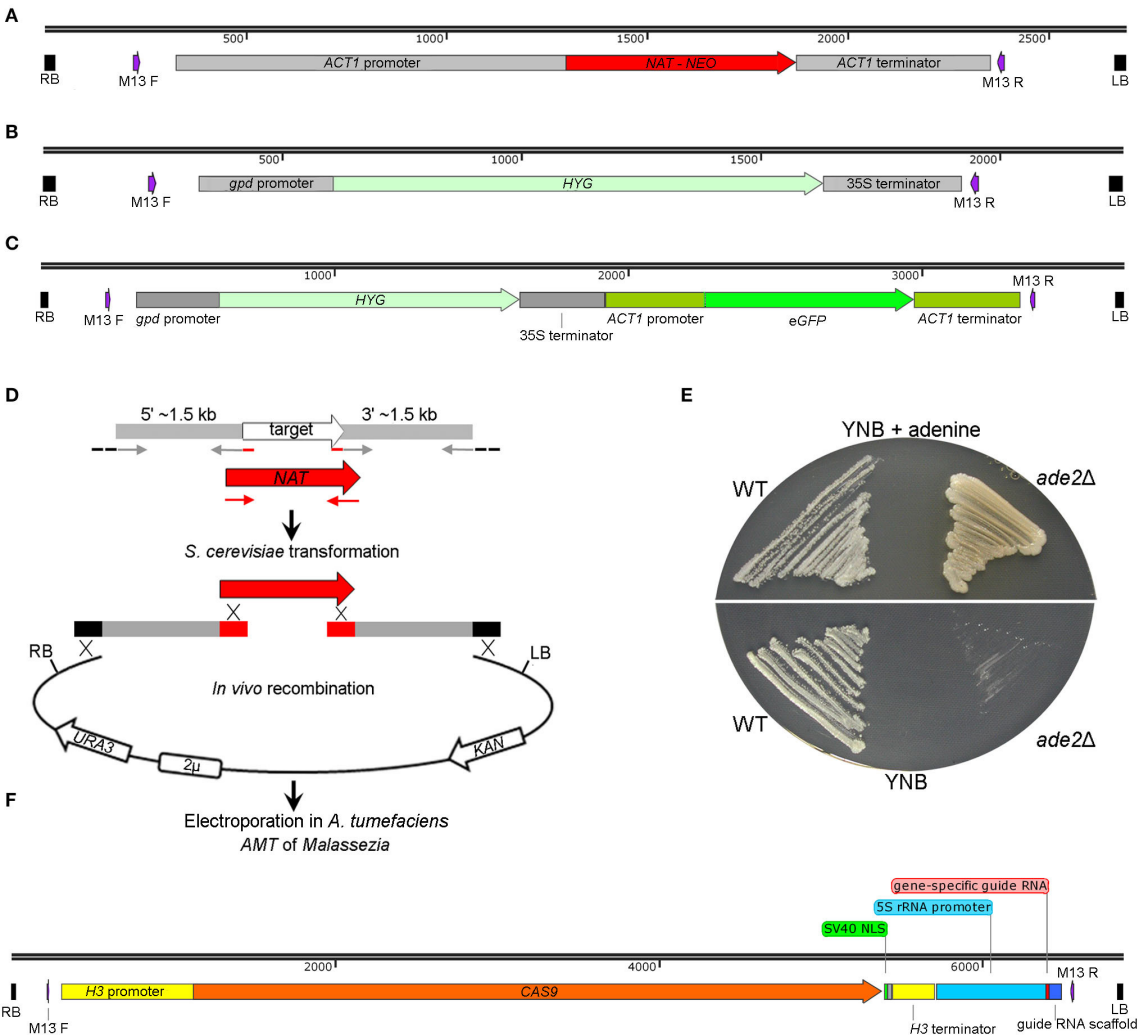
Plasmids available for *A. tumefaciens*-mediated trasformation of *Malassezia*. **(A)** T-DNA of the plasmids pAIM2 and pAIM6 conferring resistance to NAT and NEO, respectively, as reported by Ianiri et al. (2016). **(B)** T-DNA of plasmid pBHg conferring resistance to HYG, and in **(C)** the same T-DNA including also a e*GFP*-expression cassette, as reported by Celis et al. (2017). **(D)** Strategy for *in vivo* recombination in *S. cerevisiae* developed to generate plasmids for targeted gene replacement in *Malassezia;* the schematic representation is adapted from Ianiri et al. (2016). **(E)** *M. furfur ade2*Δ mutants generated through AMT in Ianiri et al. (2016); note the different growth pigmentation of the *ade2*Δ mutant compared to the WT strain on mYNB supplemented or not with adenine (mYNB stands for “modified YNB”, which includes Tween 60, Tween 20, and ox-bile). **(F)** T-DNA of the plasmid pGI40 used by Ianiri et al. (2019) for transient CRISPR/Cas9-mediated targeted gene replacement in *M. furfur*.

Several binary vectors proved to be effective for *Malassezia* transformation. Plasmids pAIM2 and pAIM6 were generated through fusing the *ACT1* promoter and terminator of *M. sympodialis* with the *NAT* and *NEO* genes to confer resistance to nourseothricin (NAT) and neomycin sulfate G418 (NEO), respectively (Figure 4A) (Ianiri et al., 2016). Celis and colleagues successfully employed plasmid pBHg that includes the *Escherichia coli hpt* gene under the control of the *Agaricus bisporus* promoter of the glyceraldehyde-3-phosphate dehydrogenase (*gpd*) gene to confer resistance to hygromycin B (HYG) (Figure 4B). Vector pBH-GFP-ActsPT further includes the *eGFP* gene from *Aequorea victoria* under the control of the *ACT1* promoter and terminator of *A. bisporus* (Figure 4C). Another vector that encodes a fluorescent marker includes a *Malassezia* codon-optimized mCherry gene fused with the *NAT* marker, as in plasmid pAIM2, through a P2A sequence (Goh et al., 2020); P2A is a 2A self-cleaving peptide derived from the porcine teschovirus-1 that was used to guarantee high expression of both the *NAT* and mCherry genes. These gene markers were also modified and reassembled to perform protein localization and chromatin immunoprecipitation (ChIP) through the generation of both N-terminal and C-terminal GFP fusion proteins, and 3xFLAG-tagged proteins (Ianiri et al., 2020; Sankaranarayanan et al., 2020).

One of the greatest advantages of AMT for *Malassezia* is its efficacy for approaches of both random insertional mutagenesis and targeted mutagenesis, which is not common for Basidiomycota fungi such as *C. neoformans* (McClelland et al., 2005). Insertional mutagenesis is carried out through AMT of a *Malassezia* species with one of the binary vectors described above, selection of stable drug-resistant transformants to be screened for a phenotype of interest, and identification of the genes that bear the random T-DNA insertion within the *Malassezia* genome; PCR-based techniques (inverse PCR and/or Splinkerette PCR) or whole-genome sequencing can be used to identify the site of insertion of the T-DNA (Idnurm et al., 2004; Ianiri et al., 2011; Ianiri and Idnurm, 2015). The random insertional mutagenesis approach was applied mainly in *M. furfur* and allowed the identification of (i) transformants unable to grow on minimal medium with T-DNA insertions in the *TYR1* and *ARG1* genes, (ii) a temperature-sensitive transformant with a T-DNA insertion in the promoter region of the *JEN1* gene, (iii) a UV-sensitive transformant with a T-DNA insertion in the *CDC55* gene, and (iv) several other transformants sensitive to the antifungal drug fluconazole, heavy metals, and cell wall stressing compounds (Ianiri et al., 2016, 2019). Insertional mutagenesis has the advantage that it can be used to discover novel genes and phenotypes; conversely, it has the disadvantage that transformants selected might have irregular and/or multiple T-DNA insertions and chromosomal rearrangements, factors that hinder the correct association between the mutated genes and the observed phenotypes. For such situations, in *Malassezia* the mutant phenotype can be confirmed through the *de novo* generation of a targeted deletion mutant for the identified gene, as we recently described (Ianiri et al., 2019).

Gene disruption mutagenesis involves the generation of a specific targeted mutant for a defined gene via homologous recombination. The first step is the generation of a gene deletion construct that includes ~ 1–1.5 kb of sequence homologous to the regions flanking the gene of interest fused with a gene marker; when recipient organisms are transformed with this allele, homology with the flanking regions allows homologous recombination and the replacement of the target gene with the gene marker. For the use of AMT for targeted gene replacement, the gene deletion allele has to be assembled and cloned within the T-DNA of a binary vector. Although this can be achieved using several approaches, for gene deletion in *Malassezia* we developed a high-throughput strategy based on *in vivo* recombination in *S. cerevisiae* to simultaneously assemble and clone the gene replacement cassette within the T-DNA of a shuffle plasmid (Ianiri et al., 2017b). Briefly, three PCR fragments that include the gene marker gene and the 1.5 kb upstream (5′) and downstream (3′) regions flanking the target genes, and the KpnI-BamHI digested pGI3 plasmid, are transformed in *S. cerevisiae* wherein endogenous recombination is enabled by homologous regions between the PCR fragments and the digested plasmid (Ianiri et al., 2016), (Figure 4D).

In our first attempt, we tested the feasibility of AMT to generate *M. furfur* targeted mutants for the *ADE2* gene, which was chosen because mutations in this gene result in a differential pigmentation compared to the WT hence allowing rapid evaluation of the results. We obtained several *M. furfur ade2*Δ mutants that displayed adenine auxotrophy and a pigmentation that varied from light pink on rich media to yellow on minimal medium supplemented with adenine (Figure 4E), which is different from other yeasts such as *S. cerevisiae* (Zonneveld and van der Zanden, 1995). Subsequently, we applied this approach to study the function of the *M. furfur* laccase-encoding gene *LAC1* expected to play a role in pathogenesis (Ianiri et al., 2016), to elucidate the mechanisms of resistance of *M. sympodialis* to calcineurin inhibitors through mutations of the *FKB1* and *MSH2* genes (Ianiri et al., 2017a), and to demonstrate that the HGT-mediated acquisition of the flavohemoglobin gene *YHB1* in *M. sympodialis* resulted in a gain of function as described above (Ianiri et al., 2020). Other mutated genes under investigation were the allergen-encoding gene MalaS8 in *M. sympodialis*, and the Rim101-alkaline pathway genes *RIM101* and *RRA1* in *M. sympodialis* and in *M. furfur* (unpublished data).

During the generation of these deletion mutants, we observed a lower rate of homologous recombination (HR) in *M. furfur*, about ~50%, compared to *M. sympodialis*, which had homologous recombination rates ranging between 90 and 100%. While the mechanisms that control the rate of HR in these fungi are unknown and worthly of further investigation, in some cases we were unable to generate targeted mutants in *M. furfur*, especially for large genes. For these reasons, a novel CRISPR/Cas9 system to increase the rate of HR and efficiently generate targeted mutants in *M. furfur* was developed. The system is based on co-transformation of *M. furfur* mediated by two *A. tumefaciens* strains to deliver both a CAS9-gRNA construct that induces double-strand DNA breaks, and a gene replacement allele that serves as a homology-directed repair template. The binary vector for Cas9 expression, pGI40, consists of the *CAS9* gene fused with the histone *H3* promoter and terminator of *M. sympodialis*, followed by the *M. sympodialis* 5S rRNA promoter fused with a gene-specific guide RNA, and a guide RNA scaffold (Ianiri et al., 2019), (Figure 4F). Using our AMT protocol, targeted deletion mutants for the *M. furfur* genes *CDC55* and *PDR10* were readily obtained with a HR rate of 100 and 83%, respectively; note that *PDR10* is large gene (~5 kb) and such a high rate of HR was achieved using shorter flanking regions of 800 bp.

## Concluding Remarks

*Malassezia* yeasts are attracting the interest of both basic and applied scientists because of their unique biological features, and importance in clinical and cosmetic settings. The availability of genome assemblies and robust tools for genetic manipulation allows both insertional mutagenesis and targeted gene replacement to be conducted. Results from these experiments can be combined with the increasing availability of transcriptomic data, with the possibility to focus further studies on novel key genes that characterize the *Malassezia* fungi. Moreover, from a more clinical perspective, tools for genetic manipulation can be combined with the use of host-pathogen interaction models, such as the easy-to-use wax moth larvae of *Galleria mellonella* (Torres et al., 2020), or a more complex murine skin model (Sparber and LeibundGut-Landmann, 2019; Sparber et al., 2019), enabling the characterization of both the fungal components that trigger skin damage and inflammation, and the inflammatory and antifungal response of the host to prevent fungal infection through immunological and molecular analyses of experimentally infected tissue.

## Author Contributions

GI and JH planned the review, read, and approved the final version. GI wrote the initial draft. All authors contributed to the article and approved the submitted version.

## Conflict of Interest

The authors declare that the research was conducted in the absence of any commercial or financial relationships that could be construed as a potential conflict of interest.
